# Cold plasma enamel surface treatment to increase fluoride varnish uptake

**DOI:** 10.1038/s41598-022-08069-4

**Published:** 2022-03-18

**Authors:** S. Fathollah, H. Abbasi, S. Akhoundi, A. Naeimabadi, S. Emamjome

**Affiliations:** 1 Faculty of Physics and Energy Engineering, Amirkabir University of Technology, P. O. Box, 15875-4413 Tehran, Iran; 2 Dental Research Center, Dentistry Research Institute, Tehran University of Medical Sciences, Tehran, Iran; 3 Department of Orthodontics, School of Dentistry, Tehran University of Medical Sciences, Tehran, Iran

**Keywords:** Biophysics, Health care, Medical research, Engineering, Physics

## Abstract

Among the available methods of enamel strengthening, fluoride varnish (FV) treatment has relatively better results. On the other hand, cold plasma technology has shown promising capacities in sterilizing the environment, surface modification, and improving adhesion. Accordingly, this study aimed to increase the adhesion of FV to the enamel surface to prolong the enamel interaction with FV with subsequently increased fluoride uptake by enamel. Emphasizing that the change in adhesion is evidence-based and has not been explicitly measured. For this purpose, we randomly divided twenty bovine teeth into two groups A (consisting of four teeth) and B (composed of four subgroups, each containing four teeth). Samples of group A and one specimen of each subset B investigated the effect of using Helium-DBD (He-DBDJ), Argon (ArJ), and Air-DBD jet on the enamel surface. Other B specimens are devoted to studying the release of FV fluoride ions from processed enamel. Two diagnostic techniques, scanning electron microscopy (SEM) and energy-dispersive X-ray spectroscopy (EDS), have been utilized to examine the samples' surface morphology and chemical analysis, respectively. Finally, the release of fluoride ions into distilled water was measured by an ion-selective electrode (ISE). SEM images showed that ArJ and Air-DBD significantly damaged enamel hexagonal structures, whereas, in the case of He-DBDJ, the hexagonal structures have only altered from convex to concave. EDX indicated an increase in calcium to phosphorus ratio and the amount of fluoride and sodium uptake on the enamel surface layer in the group processed with He-DBDJ plasma. The latter helps restore the damaged parts of the enamel. Analysis of fluoride released from the FV did not show a significant change owing to plasma processing (P ≤ 0.112). The combination of cold plasma and fluoride varnish treatment on the enamel surface might be considered as a more promising approach to increasing enamel resistance to tooth decay.

## Introduction

There are several factors involved in dental caries and related infections. Numerous studies and research in the last few decades on preventing tooth decay showed that it is still the most chronic disease in primary school children^1,2^. Minerals on the enamel surface are exchanged daily in the oral fluids. Tooth decay occurs due to carbohydrate metabolism by bacteria in the mouth, and a lower pH level than the critical value (pH 5.5), which leads to a change in the concentration of calcium and phosphate ions in saliva. The acid produced by bacteria dissolves the enamel surface and then the dentin^3–5^. It is now known that dental caries is a biofilm-sugar-dependent disease, which is minimized by the association between tooth brushing and cariogenic diet control. The use of fluorides works to reduce the progression of caries, and the best scientific evidence points to the use of fluoride toothpaste in a concentration of 1100 to 1500 ppm of fluoride, consumption of fluoridated water, and the use of sealants in permanent molars. Its action is always in a topical way. The use of additional professional fluorides is questionable. Furthermore, the use of systemic fluoride for caries control is not recommended^6,7^.

There are several ways to apply topical fluoride to the tooth surface. Topical fluoride in the form of 2% sodium fluoride, 1.23% APF gel, and FV, which is the most commonly used fluoride therapy at dental clinics^8–12^. The fluoride in these methods creates a physical barrier against the destructive effect of acid by forming a sedimentary layer of calcium fluoride on the enamel and improving tooth enamel structure by creating a fluoride storage source^13,14^. Numerous studies show that FV is superior to other forms due to its ease of use, especially for children, saving time, and the rare possibility of fluoride ingestion^15–17^. However, topical fluoride dissolves in the presence of an acid, so its protective ability is limited. Therefore, fluoride therapy needs to be repeated at regular intervals^18^. As a result, increasing the effect of fluoride coating and its durability with appropriate fluoride concentrations is still a significant challenge for researchers in this field. In this regard, the use of laser radiation^19–21^ ozone therapy^22,23^, and photodynamic therapy^24^ have been tested in this respect, although still, are not reliable.

The situation described has coincided with a plasma technology development stage that provides plasma access with two main features: atmospheric pressure and body temperature, known as cold-atmospheric pressure plasma, abbreviated CAP. Based on the mentioned features, CAP technology has been the source of numerous medical field experiments that have yielded brilliant and thought-provoking results. Due to the wide range of CAP medical applications, we avoid going into details and refer those interested to a few references for further studies^25–29^. However, let us mention some of the research related to plasma dentistry applications: plasma introduces applications in pathogens removal in bacterial plaque, tooth bleaching, bond strength, endodontic therapy, and increasing hydrophilicity^30–34^.

Among the CAP-derived effects that led us to design a research stream to strengthen tooth enamel is CAP's ability to change the surface's physical and chemical properties that CAP has processed. In other words, using CAP can be expected to alter (a) the properties of hydrophilicity and hydrophobicity, (b) change the electrical properties of the enamel surface and thus change the surface adsorption capacity, (c) change the structure of some bonds in the surface area by replacing some units in the surface structure either by CAP-derived radicals or replacement by the other agents mediated by these radicals. Accordingly, the idea that adequate processing of the enamel surface with a type of CAP could increase fluoride ion uptake and its replacement by OH bond in hydroxyapatite was worth trying. The process leads to fluorapatite formation with a stiffer structure and resistance to destructive factors and erosion. Although this dream seems achievable, its realization requires sufficient knowledge of the physical and chemical factors involved, which is possible through the design and conduct of numerous experiments.

To give an idea of the challenges ahead, we should address two main issues, the unknown plasma-enamel interaction and the diversity in the architecture of existing plasma systems. The degrees of freedom associated with both problems diversify the possible scenarios to such an extent that it is challenging to decide on the appropriate strategy. Accordingly, the pilot phase was defined to be as comprehensive as possible and, at the same time, limited to a few parameters. The analysis of the pilot results provides a degree of cognition for the choice of preferred architecture. Keep in mind that preferred architecture makes sense in fluoride uptake and minor damage caused by plasma processing to the enamel structure. Based on this, three types of helium, argon, and air were the working gas for plasma systems with three different discharge reactor designs.

## Materials and methods

### Enamel specimens preparation

This pilot study used 20 extracted bovine incisors from an abattoir in this investigation. All the methods were carried out in accordance with relevant guidelines. The guidelines are approved by the Research Committee (IR.TUMS.VSR.REC.1397.757) of Tehran University of Medical Sciences. On behalf of the Tehran University of Medical Sciences, the mentioned bovine teeth were ordered to the Tehran Slaughter Center, responsible for slaughtering cows to supply Tehran's meat under the supervision of the Ministry of Health and Medical Education. Under the personal supervision of the Tehran University, Slaughterhouse officials extracted out the bovine's teeth immediately after slaughtering the animals. Care was taken to select the newly extracted teeth to be healthy (e.g., not broken) and immediately immersed in a 0.05% chloramine solution at 4 $^\circ{\rm C}$ for four days to maintain their moisture and avoid environmental contamination. The first step in preparing the selected teeth was to thoroughly clean the soft tissues left on them with pumice slurry and hand scale. The next step was to cut the enamel with a low-speed water-cooled diamond saw (Elec-Micromotor MARATHON ESCORT II, South Korea). Next, a 10× stereomicroscope (ZSM1001 model, Zist Rahe Danesh Company, Iran) examined all samples' buccal surfaces to ensure the quality of cleanliness, absence of structural defects, and micro-fracture for removing unsuitable specimens from the collection. Then, we fixed the final 20 samples in molds (Designed and fabricated by Tehran University of Medical Sciences) containing acrylic resin (Cold-Cure Acrylic for repair, Acropars company, Iran) so that their buccal surface was upwards. Finally, they were coated with two layers of protective nail polish (NIVEA, 89536, France) to cover the enamel area leaving a treatment window of approximately 2 $\times$ 4 mm. At all sample preparation stages, their storage environment's humidity and temperature were 95% and 25 $^\circ{\rm C}$, respectively.

### Specimens grouping

After preparing 20 samples and passing them through the final quality control stage, we randomly divided them into two groups A (consisting of four teeth) and B (including sixteen teeth). We have introduced group A exclusively to study changes in enamel surface due to plasma processing. For this purpose, we first processed the enamel surface of three A group samples by Helium Dielectric Barrier Discharge Jet (He-DBDJ), Argon Jet (ArJ), and Air-DBD. Then, we compared the processed specimens with the remaining A group member, i.e., control (C). Group B, consisting of sixteen teeth in four subgroups, was assigned to a scenario in which the three subgroups' treated teeth (by three plasma devices) and the remaining subgroup's unprocessed teeth were impregnated with FV. We have identified the symbols He-DBDJ + FV, ArJ + FV, Air-DBD + FV, and C + FV at the end of this step. We used one tooth from each of these subgroups for Scanning Electron Microscope^34^ imaging and the other teeth to measure fluoride ion uptake.

### Topical fluoride application

The enamel slabs were thoroughly rinsed with distilled water and delicately dried with a fibreless napkin. As mentioned earlier, group A was only affected by plasma processing without receiving FV. In contrast, after plasma processing, the surface of Group B samples was impregnated with an FV layer containing 5% sodium fluoride (Oratech, USA) approximately 2 mm thick. The thickness of the FV layer was calculated through $m/\left(\mathrm{A \rho }\right)$ where “$A$” is the surface area of the enamel (2 mm × 4 mm), “ρ” is the FV mass density (ρ = 0.006 $\mathrm{g}/{\mathrm{mm}}^{3}$ provided by the manufacturer), and “$m$” is the FV mass ($m=0.1$ g) laid on each tooth by micro brush. According to the manufacturer's recommendation, after impregnating the samples with FV, the samples were exposed to dry air for 2 min to reduce the FV's moisture content. Finally, distilled water at 37 °C was selected to preserve the teeth. We did this in such a way that the fluoride varnish that we had previously impregnated on their surface remained intact. Distilled water is the most straightforward host for the teeth; it is easy to study the type and amount of changes in its composition. Next to the pilot phase, a possible influential factor in FV treatment that should be considered is the simulation of the oral environment with artificial saliva and its pH changes.

### SEM/EDX

Since SEM imaging uses a 10 nm gold coating layer on insulating layers such as tooth enamel, it is necessary to pay sufficient attention to the sample surface's cleanliness and dryness. The applied SEM (FEI Nova, NanoSEM, 450) operating parameters were: charge reduction mode, accelerator voltage 10 kV, working distance 4.5 mm, and a set of magnifications including 250, 500, 1000, 2000, 4000, 8000, and 15,000.

To investigate the modification in the enamel content due to the plasma process and applied FV, Energy-dispersive X-ray spectroscopy (EDX) (BRUKER, XFlash 6, 10) was utilized with operating parameters of 20 kV acceleration voltage and a magnification of 129 times. For this purpose, the window surface of 2 $\times$ 4 mm of the samples was scanned linearly by EDX to measure the specimens' elements using the reflected X-ray. EDX detector analyzes 700 to 1500 counts per second over three 20 nm spots.

### Fluoride ion release

We used an ion-selective electrode (ISE) connected to an ion meter for the fluoride analysis released from FV to aqueous solutions. At this stage, three samples of each subgroup of group B were immersed in individual falcon containers with 25 ml of distilled water at 37 $^\circ{\rm C}$ to check the ion meter's uptake of fluoride ions system (781 PH/Ion Meter, Metrohm). For the fluoride ion uptake calculation, we measured the weight of FV. To this end, it was enough to get the enamel slabs' weight at the beginning and end of the time interval. Therefore, each sample's weight was measured three times according to the standard introduced by (Company, Japan A&D) with an accuracy of 0.0001 g.

When measuring fluoride, after removing each sample from the storage container and before weighing it, we had to clean it from the storage solution attached to it at 4 $^\circ{\rm C}$. After weighing the sample, transfer it to a new container with 25 ml of fresh distilled water for the next measurement step. As described, the ion meter measured the absorption of fluoride ions at 0.5, 3, 6, 12, 24, and 48 h after plasma processing. The usual prescription to control pH and prevent the formation of fluoride complexes adds a total ionic strength adjustment buffer (TISAB). The result was a mixture of 20 ml of storage solution, 10 ml distilled water, and 10 ml of acetic buffer solution (TISAB IV, Metrohm, Ion Meter Switzerland). The fluoride concentrations measurement device was a calibrated fluoride-specific electrode (Separate electrode 6.0502.150; Metrohm) attached to an ion meter to an accuracy of 0.001 ppm. The calculated results were the fluoride number up taken per unit area of the sample in mg/cm^2^.

### Statistical analysis

One of the pilot phase statistical studies associated with the fluoride ion-release experiment was to determine the minimum number of required samples with reliable results. Since there was still insufficient evidence to justify the proposal at the start of the pilot phase, we faced severe financial constraints and managed costs carefully. In response to this necessity and with the consultation of statistics experts, and on their recommendation, PASS II software was employed using the one-way ANOVA power analysis option and specified parameters of α = 0.05, β = 0.2, and effect size = 0.95. As a result, we acquired four as the minimum number of samples for each of the 5 study groups.

To analyze the variation in FV's fluoride ion-release capability, the results of ISE, which were counted by Ion-Meter attached to it, were statistically analyzed using SPSS software (IBM SPSS statistics, version 26). Repeated Measure ANOVA was also operated to compare the fluoride ion release from FV in distilled water. To investigate whether there was a significant relationship between fluoride ion-release to the type of plasma and the time interval of each sample in distilled water, One-way ANOVA with a 95% significance parameter was used. Recall that Air-DBD, ArJ, and He-DBDJ produced the plasmas. Also, each sample was immersed in distilled water for 30 min, 3, 6, 12, and 24 h after plasma treatment and receiving FV.

### CAP devices

**Figure 1:**
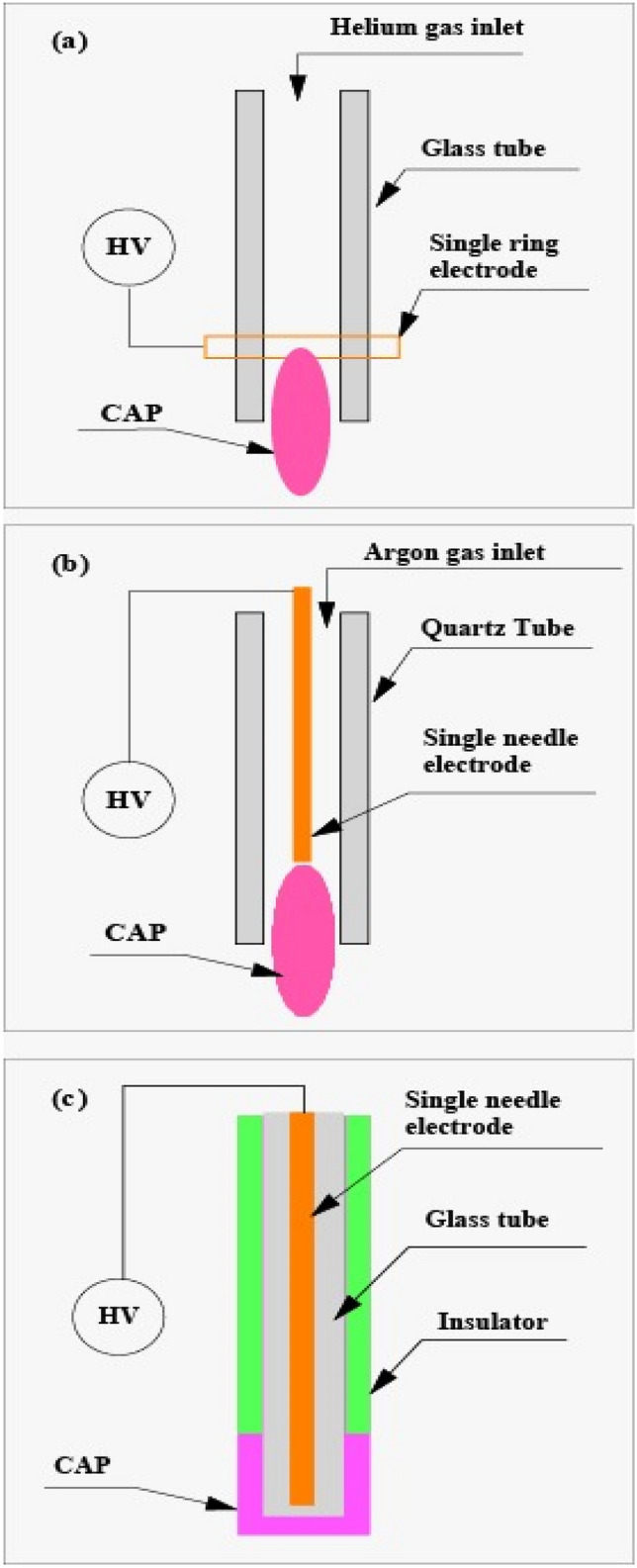
Schematic of cold-atmospheric pressure plasma devices used for enamel processing **(a)** He-DBDJ, **(b)** ArJ, **(c)** Air-DBD.

Along with our curiosity about how and to what extent cold-atmospheric pressure plasma affects fluoride ion adsorption in enamel, the variation in geometry and details of the mechanism and parameters involved in the electrical discharge process make the choice of the "appropriate architecture" for this treatment, a fundamental question^36–38^. We nominated three relatively different architectures from different strategies that could lead to selecting the appropriate architecture. We believe overlapping their features can lead to a minimum of knowledge necessary to choose the desired device. Accordingly, to better examine the results and their analysis, it must provide a clear picture of the plasma devices' geometry and characteristics in this research. Figure 1 schematically shows the three plasma generators, each of them was used independently to process every sample for 2 min.

Figure 1a shows a He-DBDJ whose main body is a glass cylinder, and helium gas is injected into one of its two ends into the cylinder, resulting in a steady flow of Q_He_ = 7 L/min. Installing a single copper ring electrode on the glass cylinder end and connecting it to the high voltage (HV) source causes the ring electrode and helium gas to interact (the discharge process). Due to the glass dielectric and gas flux, only micro-discharge is possible. The former prevents secondary electrons, and the latter regulates the interaction-time of the HV source and the base gas. The slight thermal effects of micro-discharges bring the partial plasma and carrier helium gas temperatures to about 37 $^\circ{\rm C}$. A sinusoidal HV source with a peak to peak amplitude of V_PP_ = 8 kV and a frequency of ν = 50 kHz is responsible for the electrical discharge. Since this plasma jet has only one electrode, the second (floating) electrode that plays the ground electrode's role is the tooth surface. In the experiment, the distance between the helium probe and the tooth surface was about d = 10 mm.

Figure 1b shows an ArJ. The ARG structure differs from He-DBDJ as follows. In ArJ, the reactor body (electric discharge zone) is made of quartz and has a high thermal resistance. In addition, unlike the He-DBDJ in which the retaining glass wall between the helium atoms and the high-voltage ring electrode responsible for preventing the secondary electrons' production, in the ArJ, the single needle-shaped electrode at the axis of symmetry of the cylinder is exposed to argon atoms regardless of any obstacle. That is why the secondary electrons' generation from the needle-shaped electrode and more intense electrical discharge is expectable.

Consequently, significant thermal effects (compared to the He-DBDJ) yields^36^. There are two ways to reduce the thermal effects: reducing the high voltage amplitude or increasing the argon gas flux (or both). We think it is more economical to reduce the high voltage range (less argon gas consumption, Q_Ar_ = 4 L/min). The reactor wall must protect itself from possible thermal damage while adjusting the high voltage range. Quartz is the right choice that meets the latter purpose. The parameters of ArJ applied in this study are a sinusoidal HV source with V_PP_ = 6 kV and ν = 50 kHz. Like the He-DBDJ, the second electrode is the tooth surface (floating electrode) at a distance of d = 10 mm from the argon probe.

Figure 1c is a schematic of the Air-DBD. Two main reasons justify the presence of this plasma device in the group of cold plasma generators. First, air (the air in the environment we breathe) is a base gas, and it is more cost-effective than devices that use helium or argon gases. Furthermore, the plasma generated by the electrical discharge of air adjacent to the glass tube covering the HV electrode is quiet and free of the momentum transfer caused by the base gas flux. In designing the present study's algorithm, choosing the Air-DBD without gas flux makes it possible to study gas flux's effect on the enamel surface and fluoride uptake by comparing it with two other devices. The parameters of Air-DBD applied in this study are a sinusoidal HV source with V_PP_ = 4 kV and ν = 50 kHz connected to the needle-shaped electrode. Plasma processing was performed by placing the tooth surface 1 mm from the surface of the glass tube.

## Results

We have used the Scanning Electron Microscope (SEM) and Energy-Dispersive X-ray Spectroscopy (EDX) diagnostic tools to study the effect of different plasma species on tooth enamel and subsequent changes in fluoride ion uptake and the Ion-Selective Electrode (ISE) to measure the fluoride ion release.

**Figure 2:**
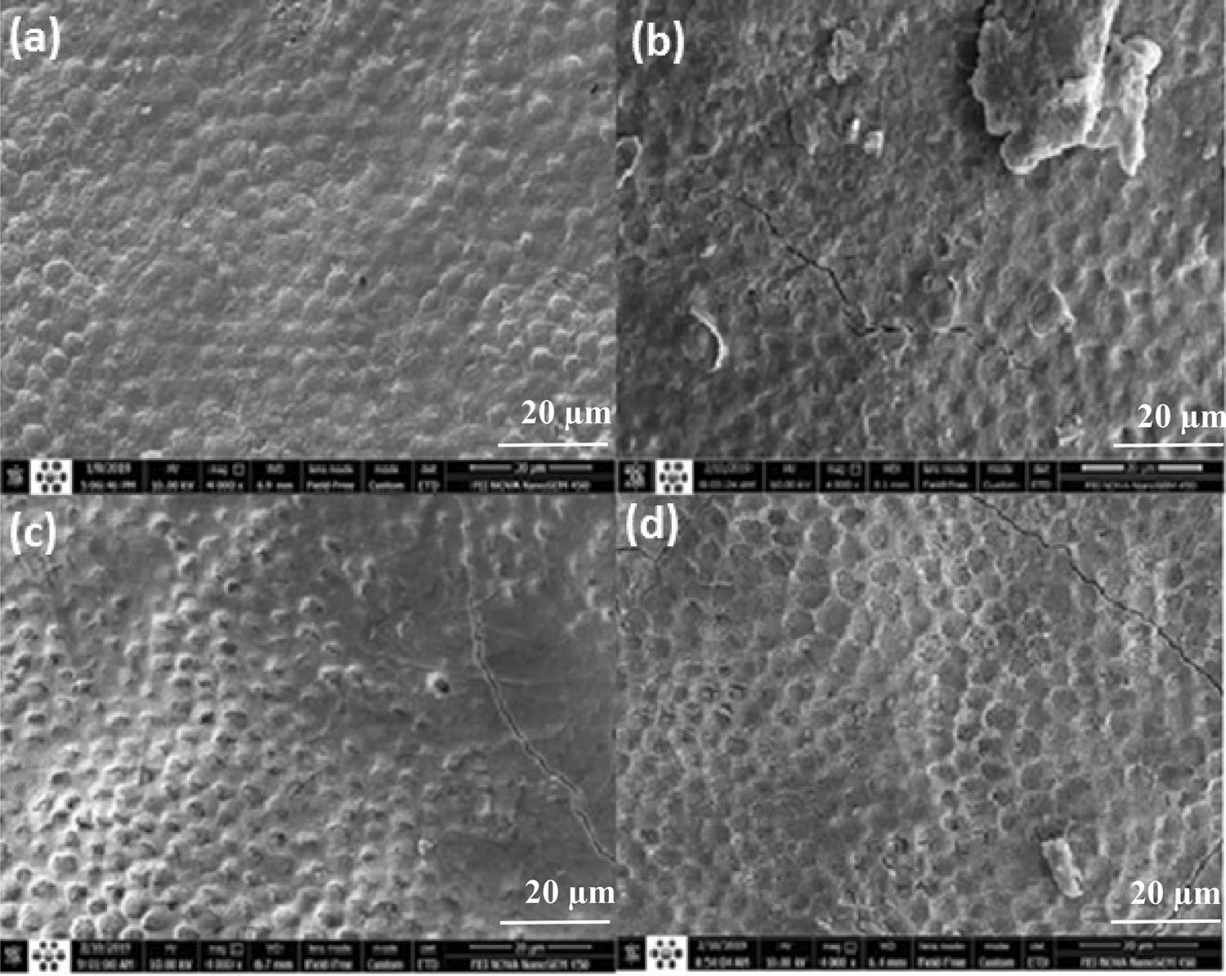
SEM images related to enamel surface treatment with **(a)** control group, process-free specimen, **(b)** Air-DBD, **(c)** ArJ, **(d)** He-DBDJ.

Figure 2 demonstrates SEM images of enamel surface treatment with (a) Control group, process-free specimen, (b) Air-DBD, (c) ArJ, (d) He-DBDJ. At a glance, the followings are observable:

1. The control sample (Fig. 2a) is distinguishable by observing porous enamel prisms and mineral deposition evidence with natural features (convex curves within hexagonal boundaries). The brighter spots are at higher altitudes than the darker ones, to our knowledge of the SEM image. Therefore, the change in image contrast is qualitatively (not quantitatively) related to mineral variation. This argument is slightly reinforced by observing Fig. 2d, in which pit-like structures are formed due to He-DBDJ and the partial erosion of humps. Of course, more reliable judgment and analysis require more accurate diagnostic tools, such as FTIR, considered in the ongoing extensive research program, following the pilot phase, devoted to investigating the He-DBDJ effects in more and accurate details.
2. The micron-sized particles in the Air-DBD processed specimen (Fig. 2b) indicate dust particles in the air.
3. Cracks are present in all samples processed by plasma (Fig. 2b–d).
4. The severe damage of hexagonal structures is observable in the samples processed with Air-DBD (Fig. 2b) and ArJ (Fig. 2c).
5. The convex structures exist similar to the control specimen in the case ArJ (Fig. 2c) and different from the control specimen with concave forms in the Air-DBD (Fig. 2b) and He-DBDJ (Fig. 2c) cases.

**Figure 3:**
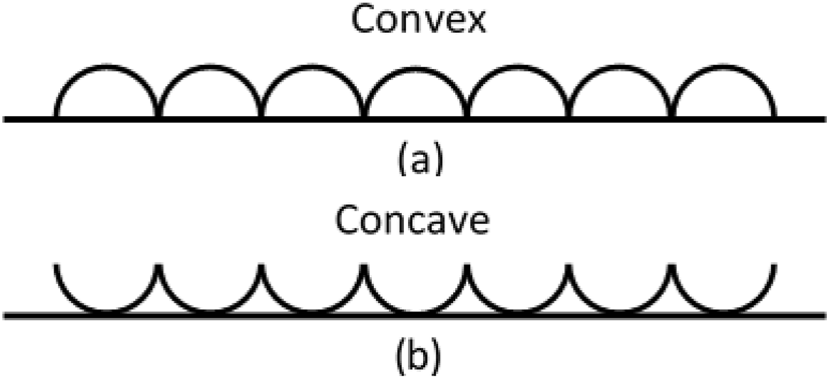
**(a)** Convex structure on enamel surface corresponding to Fig. 2a. **(b)** Concave structure on enamel surface corresponding to Fig. 2d. The rougher enamel surface is expected for FV in the concave case than the convex one.

So far, we have reviewed SEM Fig. 2 as qualitative evidence supporting the usefulness of the He-DBDJ application (Fig. 2d) in enamel surface treatment to improve FV's adhesion. To explain the word adhesion, just by looking at the SEM qualitative images, please pay attention to the diagram in Fig. 3. Figure 3 shows the convex hexagonal structure of the enamel surface corresponding to Fig. 2a and the concave structure of the enamel surface corresponding to Fig. 2d in the simplest possible form.

Perhaps this figure can be beneficial to justify the enhancement of adhesion through the use of He-DBDJ by emphasizing the difference in geometry of the two cases. The mentioned description also works well for Fig. 2b related to Air-DBD processing. Although concave structures have formed on the enamel surface due to Air-DBD, by eroding a significant portion of the enamel surface, it will eventually show less surface adhesion than He-DBDJ. By generalizing the recent discussion to the processing of enamel surfaces with ArJ, which has resulted in both convex structures and surface erosion, it is reasonable to expect minimal adhesion in this case. Nonetheless, the most relevant and easily applicable diagnostic tool that can be used to support the proposed scenario is the contact angle method, which we will perform in the program following the pilot phase.

**Figure 4:**
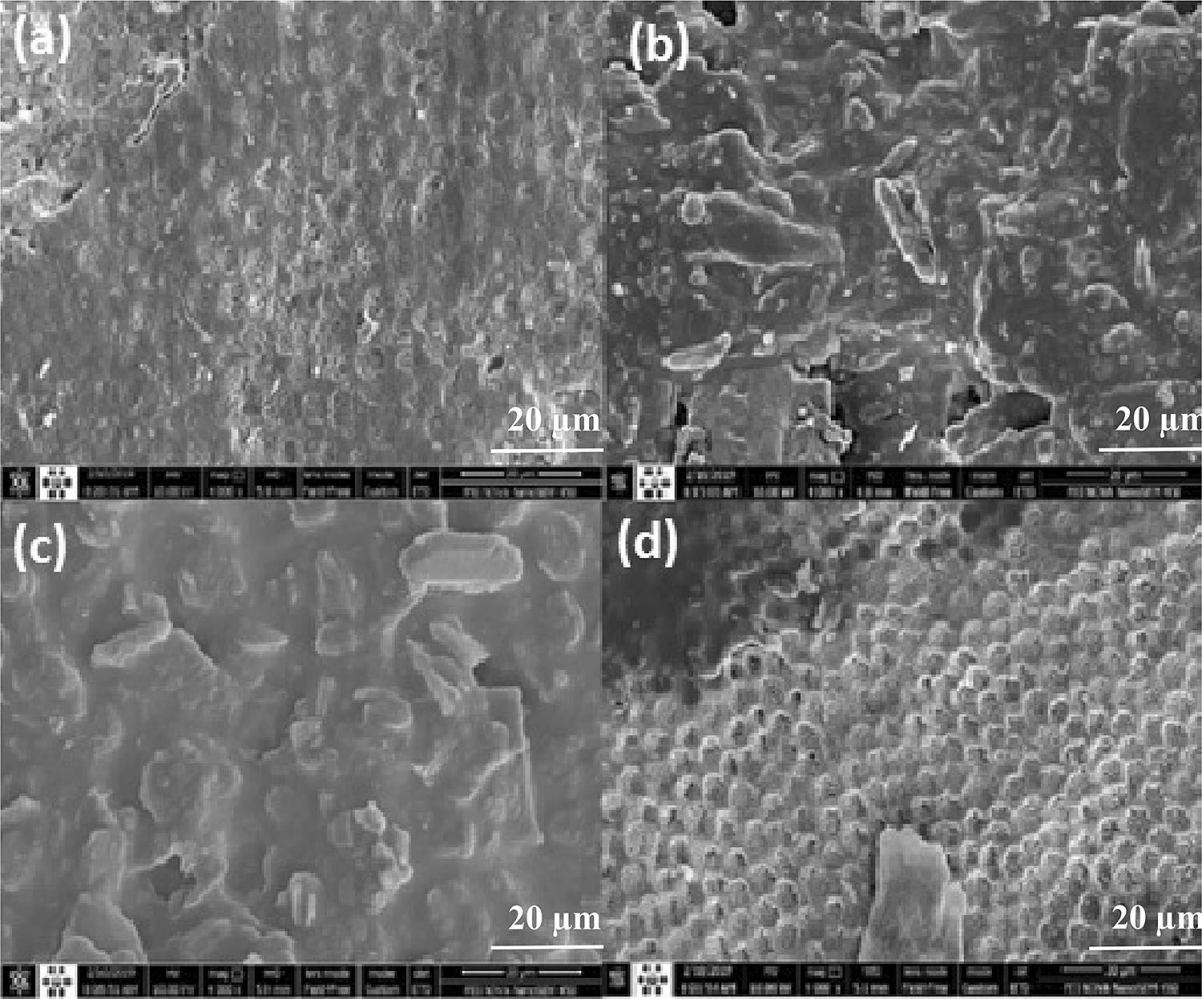
SEM images related to enamel surface treatment with **(a)** C + FV, **(b)** Air-DBD + FV, **(c)** ArJ + FV, **(d)** He-DBDJ + FV.

What can be seen in SEM Fig. 4 is the evidence that supports the presented hypotheses. Figure 4 demonstrates the processed specimens and the control one after impregnating with FV, as described earlier. The figures' arrangement, representing the control and processed specimens, is the same in SEM Figs. 2 and 4. Figure 4, regardless of the amount of dust mixed with the FV (please see Fig. 4b–d), refers to a floating FV on the surface of Fig. 4b,c specimens. Observing the hexagonal structure of the enamel surface on the outer surface of the FV (not the surface on the enamel side), Fig. 4a,d can be considered evidence that the FV is well attached to the enamel surface. In contrast, the enamel surface pattern is not recognizable on the outer surface of the FV in any of the Air_DBD and ArJ cases. We believe that the observed differences may be evidence of increased FV binding in a HeDBDJ-treated sample. Kim et al.^40^ report a similar conclusion, which is, of course, limited to comparing the He-DBDJ processed sample with the control one. In this regard, we presented a detailed analysis in the discussion section, which we hope will provide a more precise picture.

The ratio of calcium to phosphorus is one of the essential quantities in the enamel layer analysis. The larger the ratio, the greater the strength expected for the enamel structure. Emphasizing that we are only reviewing the evidence and postponing the judgment to the point where we have sufficient statistics, let us refer to another evidence that EDX has provided on the ratio of calcium to phosphorus, which can help us summarize the pilot phase. The ratio of calcium to phosphorus in different samples was C + FV 2.3, He-DBDJ + FV 2.42 (in good agreement with the report of Kim et al*.*^36^), ArJ + FV 1.51, and Air-DBD + FV 1.49. What is noteworthy is the consistency of these results with the qualitative analysis based on SEM images' viewing. The ratio of calcium to phosphorus in the two samples processed with ArJ and Air-DBD plasma is lower than the control sample, whereas the one processed by He-DBDJ plasma shows a larger ratio. In the first two, we faced the erosion of some of the hexagonal structures, while in the next two, the most important event was the transformation of the convex hexagonal structure into concave.

**Figure 5:**
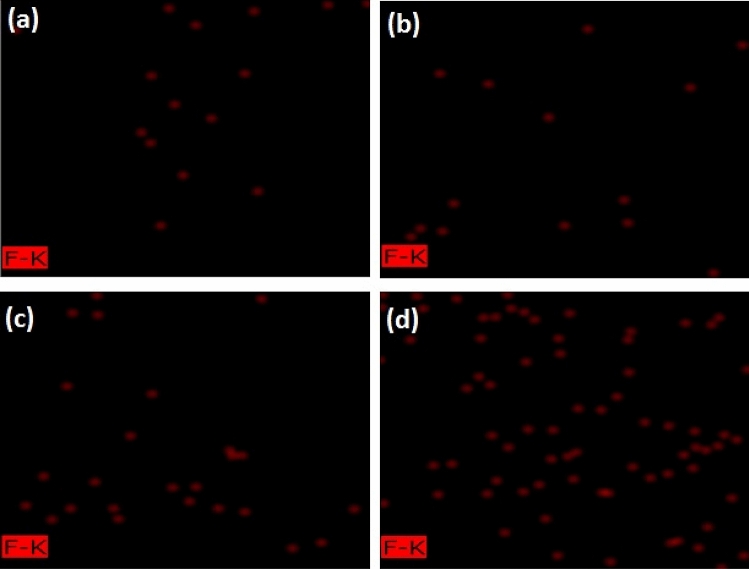
EDX mapping allows a qualitative comparison of the number of fluoride ions in tooth enamel between the control sample that received only FV and three other pieces impregnated with FV after various plasma processing. This image is the result of a 20 nm area scan of enamel. **(a)** C + FV, **(b)** Air-DBD + FV, **(c)** ArJ + FV, **(d)** He-DBDJ + FV.

The previous paragraph was not the whole story. Figure 5 shows the qualitative analysis of the EDX mapping of fluoride levels. The small number of samples (and consequently the lack of reliable statistics) does not allow us to present our observations as reliable facts confidently. So, first of all, it is necessary to re-emphasize that the presented results need to be sufficiently replicated and compared with competing techniques such as FTIR. However, even at a glance, the fluoride ions in the He-DBDJ + FV sample (Fig. 5d) appear to have increased slightly compared to other processed plasma samples (Air-DBD + FV, ArJ + FV and specially with C + FV). This result is in line with previous results that point to the benefits of He-DBDJ process.

**Figure 6:**
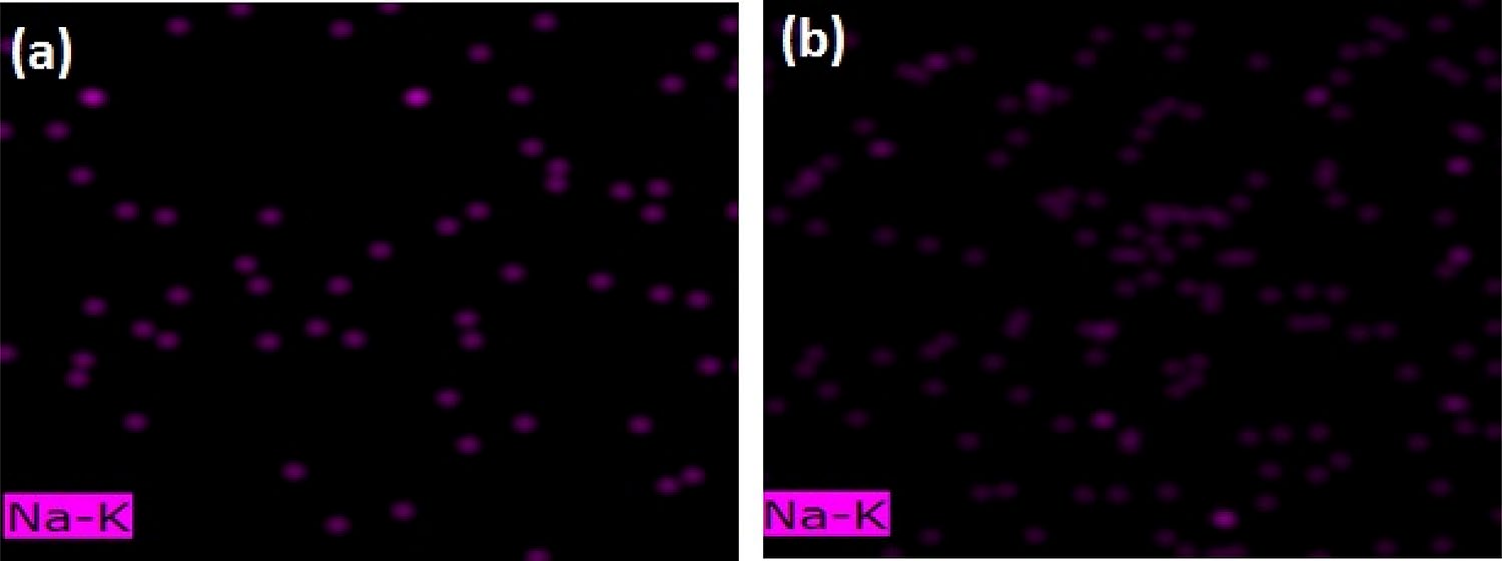
EDX mapping allows a qualitative comparison of the amount of sodium in tooth enamel between the control sample that received only FV and the one impregnated with FV after He-DBDJ plasma processing. The image is the result of a 2 nm enamel scan area. **(a)** C + FV, **(b)** He-DBDJ + FV.

Another considerable evidence in the EDX mapping analysis was sodium presence only in the control specimen and processed by He-DBDJ plasma (please see Fig. 6). We know that the source of this sodium is the FV. Nevertheless, what is interesting, and we still have no idea how to explain it, is the absence (at least in the range of used EDX accuracy) of sodium in specimens processed by the Air-DBD and ArJ plasmas.

**Figure 7:**
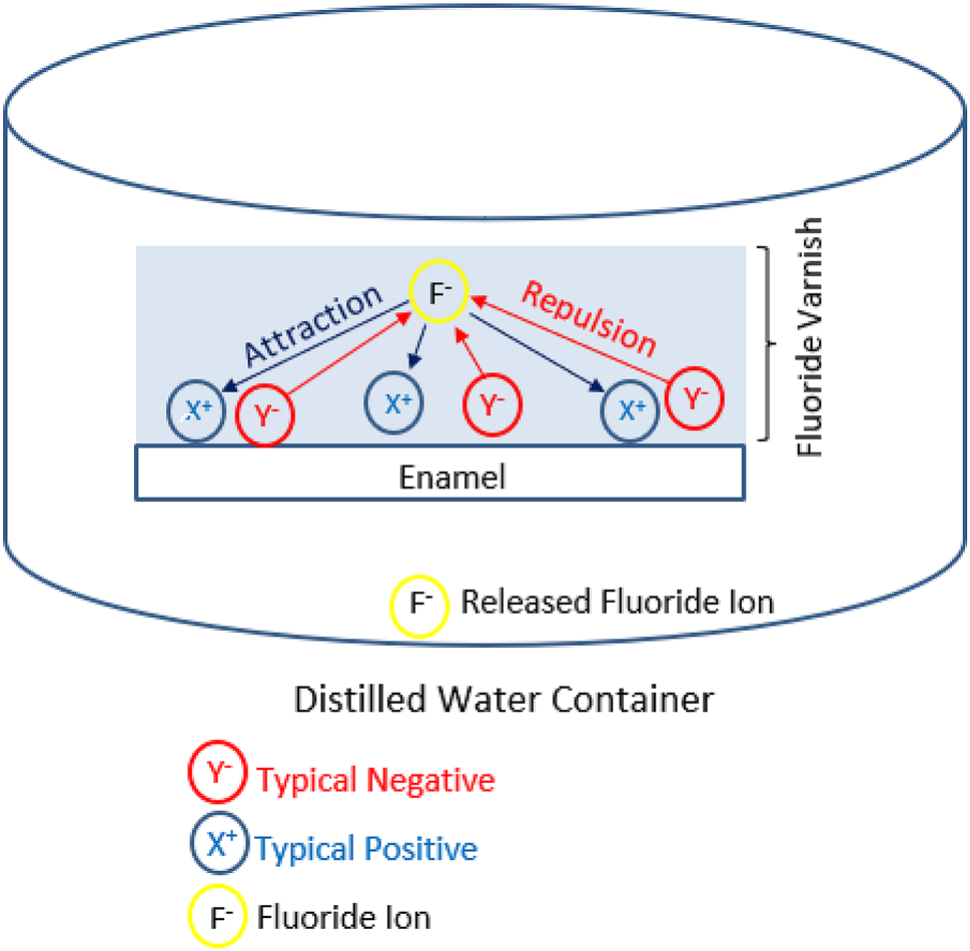
Schematic image showing the ion-release experimental idea.

Another idea that caught the group's mind about plasma processing is shown in Fig. 7. To realize this idea, we first processed the enamel surface with plasma, impregnated it with fluoride varnish, and finally immersed it in a distilled water container. In this way, we aimed to create a competition. Competition between charged plasma particles (i.e., changes in surface electrical properties) and the tendency of distilled water (associated with permanent bipolar moments of water molecules) to dissolve FV within itself, as shown in Fig. 7. Based on this algorithm, if fewer fluoride ions were measured in distilled water than the control sample, we could hypothesize that more fluoride ions were bound (within FV). Note, the only difference in the control sample preparation was the lack of a plasma therapy phase. The constraint is imposed by the charged particles' electric force (attraction + repulsion).

For this purpose, we used an ion-selective electrode (ISE) connected to an ion meter for the fluoride analysis released from FV to distilled water. At this stage, three samples of each subgroup of group B without removing fluoride varnish are immersed in individual falcon containers with 25 ml of distilled water at 37℃ to check the released fluoride ions (781 PH/Ion Meter, Metrohm).

**Figure 8:**
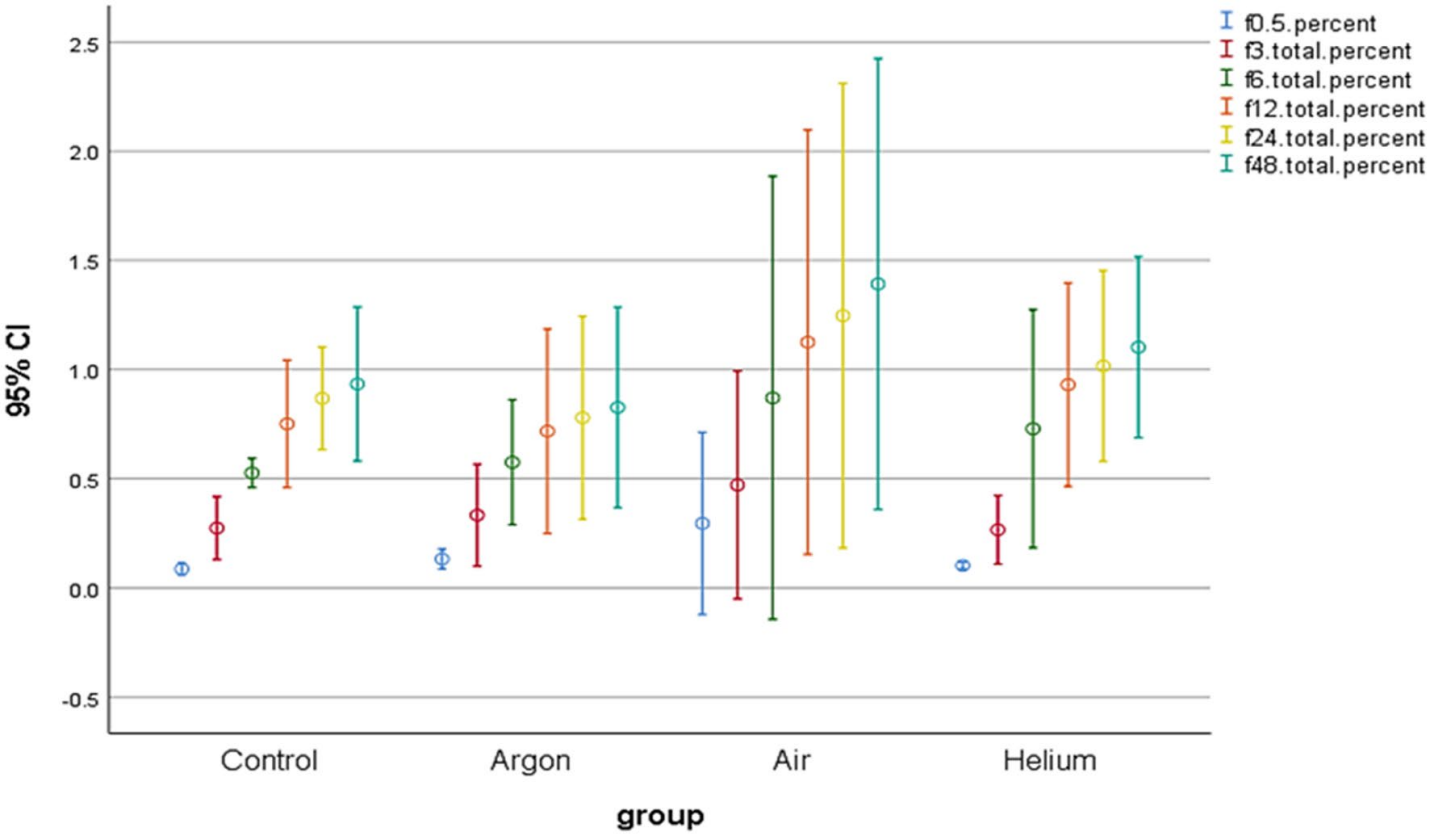
Investigation of the effect of plasma processing on the release of fluoride ions from FV. Ion meters measure the fluoride ions release at 0.5, 3, 6, 12, 24, and 48 h with P ≤ 0.112.

Figure 8 is the result of the above experiment. The results do not show a significant change. Despite what Fig. 8 confirms, it is still too early to judge plasma-derived radicals' effectiveness on the ion uptake process. Accordingly, we hope to propose mechanisms that maximize fluoride ions' maximal absorption by modifying the test method.

## Discussion

As a group dealing with CAP technology research and its applications, we knew that cold plasma, on the one hand, was prosperous in increasing dye adhesion in the textile and automotive industries. On the other hand, it has introduced a good capacity in innovative medical treatment methods. As a result, the tempting idea that CAP may increase fluoride varnish adhesion to enamel and cause more fluoride ions absorption was formed. Nevertheless, it was clear that the group faced many challenges from the beginning. In the first step, we were faced with selecting the right plasma system among the available options. That was not an easy choice.

For this reason, three options, Air-DBD, ArJ, and He-DBDJ, were nominated. In this way, it was possible to investigate the role of background gases with different mass numbers, electrodes equipped with and without dielectric, and a system containing and free of gas flux. We knew that studying the effect of enamel plasma processing is a complex matter. Therefore, for each type of plasma, at least SEM, EDX, X-ray diffraction analysis (XRD), Microhardness, Contact angle diagnostic techniques should be utilized for seven groups of teeth, each consisting of at least 19 teeth.

The cost of such research was beyond the financial capacity of the group. Therefore, to manage the cost of research and achieve a minimum of cognition, through which we are guided to select the optimal plasma system, we had to accept significant limitations in the design of the pilot phase, as presented in this report.

In selecting diagnostic systems, we prioritized SEM diagnostic. We believed that observing the morphology of the enamel surface would be a shortcut to the initial and rapid understanding of the benefits and the unknown challenges of each plasma device. In the next priority, we put the EDX diagnostic device to analyze the chemical changes of the enamel surface layer. Finally, considering the availability of an ion-selective electrode (ISE) connected to an ion meter, we designed a scenario through which we may be able to open a way to examine the adhesion of the enamel surface.

Despite all the limitations mentioned, the results were valuable enough to meet the objectives of the pilot phase described above. We selected He-DBDJ as a suitable option for the main research plane following the pilot phase. As described, the issue had many intricate details. In describing the most important of them, we used the terms "changes in adhesion" and "the rate of surface erosion." Given that no diagnostic mechanism was used to measure them, the possible ambiguity in the use of adhesion and surface erosion is quite natural. In the first step, we will discuss the ambiguity as mentioned above.

**Figure 9:**
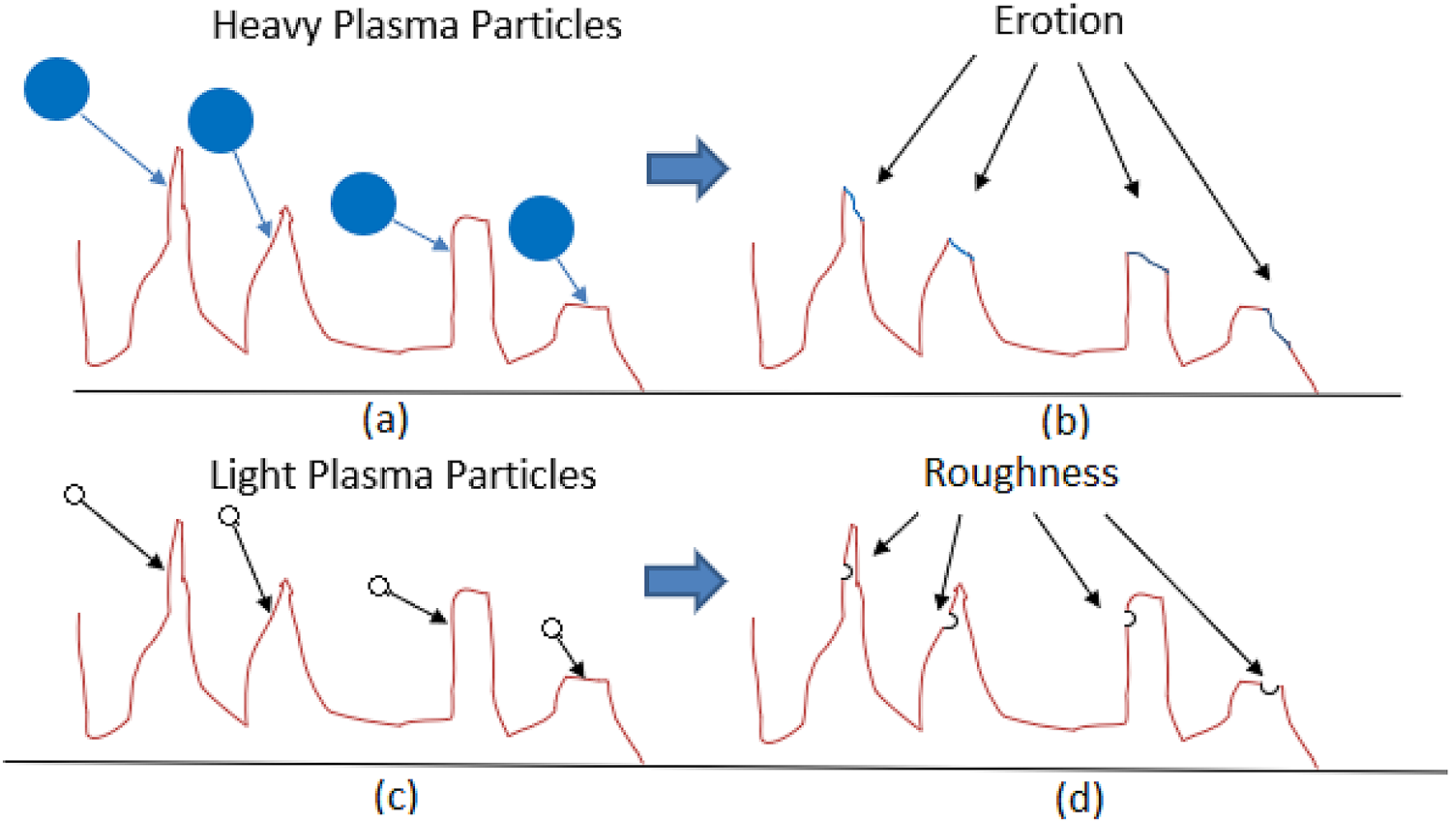
**(a)** Heavy plasma particles (Air or Argon plasma) collide with the humps of surface altitude fluctuation. **(b)** Heavy plasma particles erode the bumps and smooth the surface (erosion). **(c)** Lighter plasma particles (Helium plasma) collide with the humps of surface altitude fluctuation. **(d)** After colliding light plasma particles with the bumps, they create pits of their size in the humps, which causes more surface roughness.

To clarify the associated hypothesis supporting the physical mechanism, please see Fig. 9. Figure 9 shows a schematic of the enamel surface on a scale with visible surface fluctuations (red curve). The solid blue circles (in Fig. 9a) represent the heavy plasma particles from Air-DBD or ArJ compared to the light plasma particles from helium He-DBDJ represented by the hollow black circles (Fig. 9c). Arrows attached to each circle simulate the direction of particle momentum. As shown in Fig. 9b, part of the surface bumps is worn through the collisions with heavy plasma particles. Therefore, a surface with lower altitude fluctuations (smoother, more slippery, and less adhesive) is created, marked in the form of smoother peaks (the blue one). The impulse force is proportional to the changes in momentum, and momentum is proportional to the particle mass. Thus, we expect to encounter smoother peaks due to Air-DBD and ArJ. The reduced friction coefficient of the enamel surface is the macroscopic interpretation of this scenario. However, lighter plasma particles (from He-DBDJ) hit the bumps with less force due to their lower mass (in Fig. 9c), and dig a hole similar to helium size is created in the bumps in the most effective collision (Fig. 9d). Note that the altitude fluctuations do not alter due to the collision scenario with lighter plasma particles. However, some holes are added to the altitude structure, leading to more surface roughness and adhesion.

Consequently, the adhesion of FV to the surface of those samples processed by Air-DBD and ArJ was reduced. Instead, the control sample and the He-DBDJ processed sample (with a relatively complete hexagonal structure) show recognizable hexagonal structure even after applying the varnish.

In studying SEM photos, we noticed cracks on the enamel surface, which was one of the unknown challenges for us. With sufficient caution, it may be possible to link the presence of cracks to plasma processing. Because it is visible in all processed samples but not in the control sample. However, our statistics at this stage do not allow us to judge cracks before plasma processing. Nevertheless, if this is the case, it could be due to the transfer of momentum of the plasma particles to the enamel crystal or the fields' interaction around the discharge produced radicals with the enamel layer. The particles' momentum has a transition part supplied by the carrier gas flux and another part due to the acceleration of charged particles in the HV field. The latter has a random velocity distribution due to collisions with other particles that often occur at atmospheric pressure. We know that the enamel mass does not have a homogeneous structure (that if it did, it would be better to use the word enamel crystal instead). In fact, it consists of several different crystals (such as hydroxyapatite, fluorapatite, different types of Hydroxycarbonate apatite), which stuck together^39,40^. The bonding forces within crystal constituents are far more substantial than the forces that hold various crystals together. Accordingly, the enamel layer's weakness is the heterogeneity boundaries (the typical boundaries of two or more crystalline species). The most likely place for cracks to form and spread is in a heterogeneous area-the neighborhood of two different crystals (See Ref.^35^ for a research study on enamel cracks). We do not know how to check the cracks by SEM because access to the control sample is lost due to a thin layer of gold coating. However, in the main research plan, we will dedicate a part to it, provided that we can introduce a diagnostic method for cracking.

Significant erosion of the enamel surface in both Air-DBD and ArJ reinforces the hypothesis that the carrier gas flux cannot be considered a substantial factor in the erosion process. Because of QHe > QAr and QAir = 0. The main agent's search makes accurate spectroscopy of a distance of one centimeter from the He-DBDJ, ArJ, and one millimeter from the Air-DBD inevitable. Adequate knowledge of the different types of radicals produced and their populations will significantly contribute to deciphering the erosion factors. However, even with this amount of information, it can be assumed that the atomic mass of carrier gases has a meaningful relationship with the amount and manner of erosion. Since the highest amount of enamel surface erosion Air-DBD (mO2 = 31.998, mN2 = 28.014) and then ArJ (mAr = 39.948) and finally the least that has occurred by converting convex structures to concave in He-DBDJ (mHe = 4.002602)^41,42^. Whether these gases themselves directly change the enamel surface after ionization or produce a chain of oxygen and nitrogen radicals should be left to post-spectroscopy.

In addition to the benefits that plasma processing may play in increasing fluoride uptake, undesirable side effects such as the possible involvement of plasma processing in the presence of cracks and erosion of the enamel surface should not be neglected. One of the purposes of the main research plan is to quantify these adverse effects in a way that allows one to judge whether plasma processing can be used as a treatment.

Sodium presence in the EDX mapping analysis is noticeable. The presence of sodium can temporarily increase local pH. The temporary increase in pH provides the conditions for the chemical reaction of calcium and phosphate ions in the enamel and calcium phosphate formation on the tooth surface. As this process continues, the layer is converted to hydroxycarbonate apatite, structurally and chemically similar to biological apatite^37^. In other words, the presence of sodium activates mechanisms that play a beneficial role for the enamel layer by forming hydroxycarbonate apatite. As was shown, the amount of sodium in the He + FV sample is higher than in the C + FV sample. Therefore, in terms of the presence of sodium and its subsequent mechanisms, it seems that performing FV after processing enamel with He-DBDJ plasma will be more effective than other techniques in this study.

Putting together the puzzle pieces reported in this paper leads us to the idea that if plasma processing increases FV uptake into the enamel layer (which must be precisely and in detail quantified), He-DBDJ is a promising choice in comparison to other plasma devices used in this study.

## Conclusion

Within the limitations the present study, the following conclusions may be drawn:

1. Although SEM images indicated that Air-DBD and ArJ damaged the hexagonal structure of sample, He-DBDJ did not affect significant morphology change on these dental substrates.
2. He-DBDJ + FV significant increase in fluoride ions in the sample of compared to other plasma processed specimens.
3. The ion release from varnish fluoride of enamel in distilled water results do not show a significant change in compared groups.
